# Dental Materials Design and Innovative Treatment Approach

**DOI:** 10.3390/dj11030085

**Published:** 2023-03-17

**Authors:** Francesco Gianfreda, Patrizio Bollero

**Affiliations:** 1 Department of Industrial Engineering, University of Rome “Tor Vergata”, 00133 Rome, Italy; 2 Department of System Medicine, University of Rome “Tor Vergata”, 00133 Rome, Italy

In recent years, technological innovation has had exponential growth, resulting in positive implications in dentistry.

In the field of implantology, the development of bioactive surfaces and biomaterials has made it possible to enhance the early biological response [1,2,3,4].

In addition, Gianfreda et al. showed that from a microscopical point of view different bioactive surfaces react differently to decontamination processes. This also leads to a different healing ability around implants with peri-implantitis treated with different decontamination systems [5].

Similarly, the use of biomaterials as scaffolds for bone regeneration has undergone an incredible development in recent years. One of the most innovative aspects is certainly the use of demineralized dentin as autologous graft material [6,7,8]. According to some authors, BMPs may be present in these grafts and could have an osteoconductive effect [9].

In the field of prosthetics, the development of CAD-CAM technologies and the advent of intraoral and facial scanners have allowed a faster and increasingly tailor-made approach, thanks to the use of milled titanium bars and milled ceramic materials [10,11,12,13,14].

In the field of orthodontics, the advent of transparent aligners has drastically changed the approach to the patient in the developmental age. In fact, aligners allow you to obtain predictable and fast results without compromising the patient’s aesthetics [15].

This Special Issue is concerned with all aspects of all dental specialties dealing with this topic. There is an important question that we can ask ourselves: what are the most interesting materials in all of the sub-fields of dentistry? How can the latest generation’s materials and technologies influence the patient care plans?

## Data Availability

Not applicable.
